# Body tactile stimulation intensifies appeasement behavior in a cichlid fish

**DOI:** 10.3389/fnbeh.2026.1821679

**Published:** 2026-08-20

**Authors:** Bianca Cambiaghi e Silva, Ana Carolina dos Santos Gauy, Sarah Garcia Prado, Manuel Gesto, Marta C Soares, Eliane Gonçalves-de-Freitas

**Affiliations:** 1 Departamento de Ciências Biológicas, Universidade Estadual Paulista (UNESP), São José do Rio Preto, Brazil; 2 Centro de Aquicultura da UNESP, Jaboticabal, Brazil; 3 DTU Aqua, Section for Aquaculture, Technical University of Denmark, Hirtshals, Denmark; 4 CIBIO, Research Centre in Biodiversity and Genetic Resources – InBIO Associate Laboratory, University of Porto, Vairão, Portugal; 5 BIOPOLIS Program in Genomics, Biodiversity and Land Planning, Centro de Investigação em Biodiversidade e Recursos Genéticos, Vairão, Portugal; 6 CIBIO, Research Centre in Biodiversity and Genetic Resources, InBIO Associate Laboratory, School of Agriculture, University of Lisbon (ISA), Lisboa, Portugal; 7 MARE–Marine and Environmental Sciences Centre/ARNET–Aquatic Research Network, Institute for Research and Advanced Training (IIFA), Universidade de Évora, Évora, Portugal

**Keywords:** aggression, fish welfare, sensorial enrichment, social touch, subordinate behavior

## Abstract

Social touch, provided by body tactile stimulation (TS), is known to trigger appeasement behavior and social stability in mammals. Recent studies have shown that TS also plays a role in regulating social interactions in fish by reducing aggression between individuals. While this effect may stem from decreased aggression in dominants, the potential contribution of increased appeasement behavior in subordinates has been overlooked. Here, we investigated whether TS facilitates the onset of appeasement behavior in the cichlid fish *Geophagus iporangensis*, a species that exhibits clear dominance-subordination relationships. Isolated individuals were assigned to one of two treatments, either with TS (provided by plastic sticks lined with silicone bristles) or without it for 21 days. Then, each fish was paired with a socially experienced dominant individual to enable rapid hierarchy resolution. Initial appeasement scores did not differ between treatments. However, individuals who experienced TS exhibited significantly higher appeasement scores during the final 5 min of the contest than the control group. Additionally, fish under TS showed an increase in the appeasement score after 10 min of agonistic interaction, while it did not change in the treatment without TS. Overall, this study provides the first evidence that TS can mitigate aggressive interactions by intensifying subordinates’ appeasement behavior, thereby affecting social dynamics similarly to mammals.

## 1 Introduction

The social behavior of teleost fishes is diverse and includes the formation of groups organized in schools, territories, dominance hierarchies, and even more complex forms, such as cooperative breeding (Taborsky, 2016). Among teleosts, the Cichlidae family has a rich behavioral repertoire and complex social interactions that vary according to reproductive behavior (Barlow, 2000). Aggressive interactions are part of the social behavior of cichlids and occur mainly during the establishment of social hierarchies (Satoh et al., 2019) that occur between two or more individuals of the same species and encompass aggressive and submissive behaviors (Wazlavek and Figler, 1989). In this sense, aggressive behavioral units correspond to the actions of attacks (overt fights) or threatening (restrained fights) that communicate a greater aggressive motivation to escalate a fight (Young, 2019). Submissive behavioral units, in turn, refer to actions that communicate a reduced motivation to escalate a fight against a dominant individual or one with greater fighting ability (Petit, 2010), reducing the likelihood of aggression from other individuals (Ruberto et al., 2020). Following the establishment of social ranks, the frequency of overt fights decreases and signaling (i.e., displays) increases.

Dominant and subordinate individuals typically communicate their social position through visual stimuli, by changing posture and body color (Barlow, 2000), along with chemical (Keller-Costa et al., 2015; Gonçalves-de-Freitas et al., 2008), acoustic (Nelissen, 1978; Amorim et al., 2019), and tactile communication (Butler and Maruska, 2015, 2016a). The maintenance of dominant social positions can depend on a range of behaviors, such as threats and attacks, as well as an individual’s ability to communicate their social status through signals that provide information about their dominance rank (Tibbetts et al., 2022). For example, dominant males of Mozambique tilapia (*Oreochromis mossambicus*) store and use urine as a “dominance pheromone” to modulate opponents’ aggression and stabilize the hierarchy (Barata et al., 2007). On the other hand, subordinate fish can also communicate their reduced motivation to escalate a fight against a dominant individual or one with greater fighting ability by exhibiting submissive behaviors (Petit, 2010). Therefore, to live in a dominance-based hierarchy, individuals must communicate not only their social position to others but also signal their aggressive “intentions,” which helps to stabilize the hierarchy and promote group coexistence without starting a fight (Frommen, 2020). This motivation can be based on evaluating the opponent’s abilities compared to the individual’s own (Elwood and Arnott, 2012). In this sense, although various factors are involved in individual aggressiveness, the level of aggressive events in a fight depends on both the aggressive behavior of the dominant and the appeasement behavior of the subordinate. Therefore, to understand the dynamics of social organizations, it is essential to consider all strategies used by animals to manage their conflicts (Aureli et al., 2002).

Appeasement behavior is a set of non-aggressive signals and is hypothesized to reduce aggressive behavior in the receiver (Firnkes et al., 2017). The more complex the social interaction is, the more complex is the appeasement behavioral repertoire (Judge and de Waal, 1993). In fish, although behaviors such as helping can act as appeasement signals (Bergmüller and Taborsky, 2005), submission is usually considered as part of appeasement (Bergmüller and Taborsky, 2005). In fact, submissive communication reduces aggressive propensity in the receiver (Reddon et al., 2021), therefore, it is within the appeasement behavior definition. According to Reddon et al. (2021), submissive behaviors can be divided into avoidance behaviors and submission signals. Avoidance behaviors, such as defensive postures and fleeing, primarily enable animals to evade aggression directly and function secondarily as a communication of submission (Reddon et al., 2021). Submission signals, on the other hand, primarily act as communication; for example, changes in body coloration (Reddon et al., 2021). In this sense, we consider the whole submissive repertoire as appeasement behavior because it reduces the likelihood of aggression from other individuals (Ruberto et al., 2020).

Appeasement behavior is beneficial for both fish involved in the fights, as aggressive interactions will require high energy costs for the individuals (Copeland et al., 2011). Some of the known signs of submission in fish that promote appeasement are head-up signals (Ruberto et al., 2020), iris darkening (Miyai et al., 2011) and changes in body color (Barlow, 2000). Studies of *Neolamprologus pulcher*, a highly social species, have shown that this species exhibits great social motivation (Balshine et al., 2017) and is more likely to resolve conflicts by using submission signals (Hick et al., 2014) compared to a non-social species. These findings suggest that social motivation and conflict resolution are linked to social complexity in ways that are significant for the evolution and maintenance of sociality (Balshine et al., 2017; Hick et al., 2014). However, appeasement remains understudied in fish, with studies on the behavior of dominant individuals in the context of aggression and fight resolution being more common.

Despite aggression being part of the natural behavior of cichlids, some rearing conditions in artificial environments, such as aquariums or tanks, can increase aggressive interactions to non-adaptive levels (Gonçalves-de-Freitas et al., 2019). In this context, environmental enrichment has been used to reduce the impact of artificial environments on fish welfare (Arechavala-Lopez et al., 2022), including some for cichlids (Torrezani et al., 2013; Kadry and Barreto, 2010; Barley and Coleman, 2010). Tactile stimulation, for example, can act as a sensory enrichment that reduces aggression as well as social stress and energy expenditure of fighting in fish (Bolognesi et al., 2019; Gauy et al., 2022, 2023; Soares et al., 2011).

Tactile body stimulation is a sensory experience resulting from physical contact between two or more individuals in natural settings or by artificial apparatuses (Bolognesi et al., 2019). On coral reefs, for instance, some fish species, called “clients”, visit other fish, usually smaller ones called “cleaners”, which remove parasites from the clients’ bodies, but also provide a massage-like tactile stimulation (Bshary and Côté, 2008). It has been later shown that these massages, artificially done by a cleaner fish model, reduce stress in client fish (Soares et al., 2011). In fact, the tactile stimulation involved in the client-cleaner interaction is hedonically positive for clients and is mediated by the opioid system, as it is in humans (Maximino et al., 2025).

Besides the positive effects on client-cleaners’ mutualistic interactions, studies by our research group have shown that artificial tactile stimulation (provided by an apparatus with silicone bristles) also generates positive effects on Nile tilapia. This is an African cichlid species widely used in aquaculture and whose social behavior is based on a territorial dominance hierarchy (Gonçalves-de-Freitas et al., 2019). When the fish were exposed to tactile stimulation for 7 days, they exhibited more restrained fights (threats) and less overt fights compared to the control, showing that tactile stimulation reduces aggression in these individuals (Bolognesi et al., 2019). In addition, the long-term stimulation also increases growth rate (Gauy et al., 2022), reduces the effects of high stocking density (Gauy et al., 2023), and markedly lowers aggression in Nile tilapia social groups. Gauy et al. (2021) also demonstrated that male Nile tilapia that used a TS device more times became subordinate in further contests, suggesting that TS may promote appeasement behavior by leading fish to become subordinate. Here, we investigated the effect of tactile stimulation on the appeasement behavior of the cichlid fish *Geophagus iporangensis*, a species that exhibits evident dominance-subordination relationships (Miyai et al., 2011). We predicted that tactile stimulation would intensify the appeasement behavior of *G. iporangensis*, probably accelerating social hierarchy resolution. Furthermore, by accelerating rank establishment, subordinates may become less stressed.

*Geophagus iporangensis* Haseman 1911 is a native cichlid fish of the Ribeira de Iguape River basin (Oyakawa and Menezes, 2011), which exhibits a complex substrate environment structure characterized by submerged logs and branches (Cetra et al., 2022). This species exhibits substrate-sifting feeding behavior to obtain benthic invertebrates (Lopez-Fernandez et al., 2012), which involves ingesting substrate (either sand or silt) and filtering out edible particles (Lopez-Fernandez et al., 2012). In relation to reproduction, cichlid species exhibit monogamous behavior with biparental care, involving elaborate reproductive mechanisms that range from mate selection and courtship to territory establishment and defense (Barlow, 2000). Like other *Geophagus* species, *G. iporangensis* may employ diverse reproductive strategies including nest construction on elevated surfaces (e.g., *Geophagus brasiliensis*) or oral incubation (e.g., *Geophagus steindachneri*), where females carry eggs and developing fry in their mouths for approximately 2 weeks (Wimberger, 1992). Furthermore, cichlid species organize into social hierarchies and show territorial behavior (Barlow, 2000), which involves frequent physical interactions during confrontations and hierarchy establishment. In this sense, in their natural habitat, *G. iporangensis* may experience tactile stimulation when swimming between vegetation, rocks, and the substrate during foraging, or even when engaging in activities involving physical contact, such as courtship, nest preparation, and territorial disputes. However, despite the rich behavioral repertoire and complex social interactions of cichlids species, specific interactions with their natural environment, such as tactile stimulation, remain understudied.

## 2 Materials and methods

### 2.1 Animals and housing

Adult *G. iporangensis* specimens from the Aquaculture Centre of UNESP (CAUNESP) were acclimatized in polyethylene water tanks (ca. 500 L, 1 fish/10 L) at 28°C, a light regime from 7:00 a.m. to 7:00 p.m. and fed once a day with food for tropical fish (28% CP) to apparent satiety. Water quality was maintained by biological filters (400 L/h) and constant aeration. Tanks were siphoned weekly to remove fecal and food leftovers, with no more than 25% of the total water volume removed to minimize dilution of possible social chemical signals in the water (Gonçalves-de-Freitas et al., 2008; Gauy et al., 2018). Six specimens were deposited in the Ichthyological Collection of the Department of Biological Sciences, UNESP, São José do Rio Preto (DZSJRP 23338).

### 2.2 Experimental design

**FIGURE 1:**
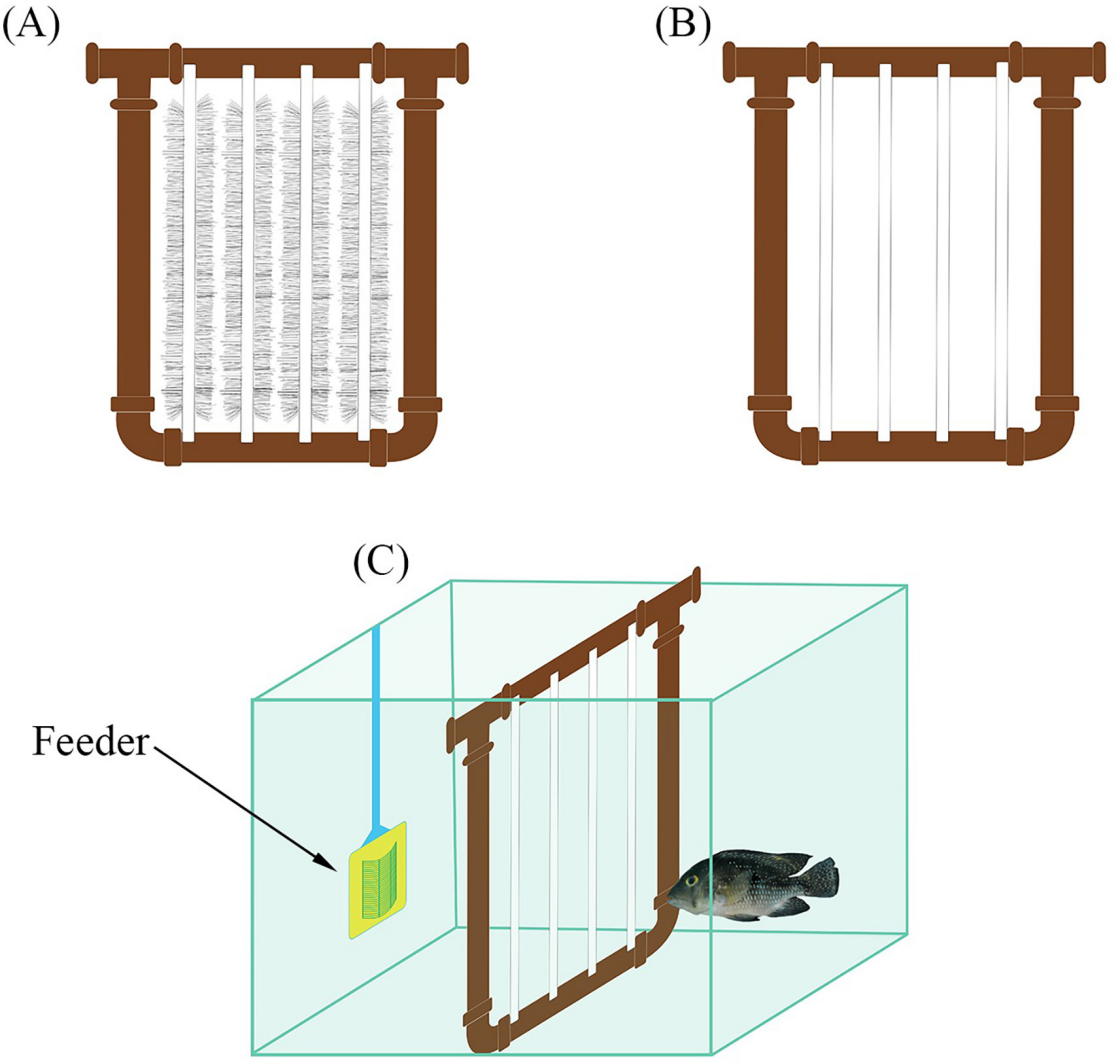
Setup diagram. Tactile stimulation apparatus with silicone bristles **(A)** and the control apparatus without silicone bristles **(B)**, positioning of the apparatus **(C)** in the center of the aquarium. The feeder consisted of a plastic stick with a plastic net attached to the end to hold the food in the aquarium.

Isolated individuals of *G. iporangensis* without sex distinction were kept for 21 days in one out of two groups: one that provided TS (*n* = 13) and the other without TS (*n* = 13) as a control group (Figures 1A–C). Then, to test whether tactile stimulation would favor the appearance of appeasement behavior, each individual was paired with another one of a larger size without sex distinction and with prior dominance experience (see below). We also evaluated fish stress levels, as TS can attenuate stress in some fish species (e.g., Soares et al., 2011). Due to the lack of external sexual dimorphism in this species, the analysis of sex differences is not included in this study.

Fish weight and standard length (mean ± SE) were as follows: TS group - 22.12 ± 2.46 g and 8.59 ± 0.27 cm; group without TS - 23.11 ± 2.02 g and 8.59 ± 0.25 cm. They were tested in aquariums of 40 × 30 × 40 cm; ca. 48 L, with an external filter, gravel substrate, water maintained at 28°C, and a light regime from 7:00 a.m. to 7:00 p.m. pH levels were monitored and were kept at 7 (measured with the electronic device Hanna HI98127). The aquariums were covered on all sides with opaque blue plastic to prevent visual contact with animals from neighboring aquariums and with the researchers.

### 2.3 Tactile stimulation protocol

Tactile stimulation was provided by an apparatus previously developed in our laboratory, similar to that described in Bolognesi et al., 2019 and Gauy et al., 2022. It consists of a rectangular PVC frame with vertical plastic sticks and silicone bristles on their sides (Figure 1A). An apparatus without the silicone bristles was used as a control (Figure 1B). In both cases, the device was placed in the middle of the aquarium (Figure 1C). In the TS group, the fish received tactile stimulation as they passed between the bristles; in the group without TS, the fish was able to pass freely through the device, without tactile stimulation. The animals were filmed (the camera was positioned above the aquariums) daily for 10 min before, 10 min during and 10 min after feeding, both in the morning (9 a.m.) and afternoon (2 p.m.) to minimize circadian influences. The food (soft processed meat) was placed inside a feeder introduced at the opposite end of the aquarium to where the fish were (Figure 1C), so that the fish experienced body TS when passing through the apparatus to access the food. The feeder was manually introduced after 10 min of recording and then removed again 10 min later. The frequency of crossings through the apparatus in the two groups was quantified from the recordings only before and after feeding (40 min total: 20 min before feeding and 20 min after feeding across both time periods) to avoid data biases.

### 2.4 Characterization of agonistic interaction of *G. iporangensis*

**FIGURE 2:**
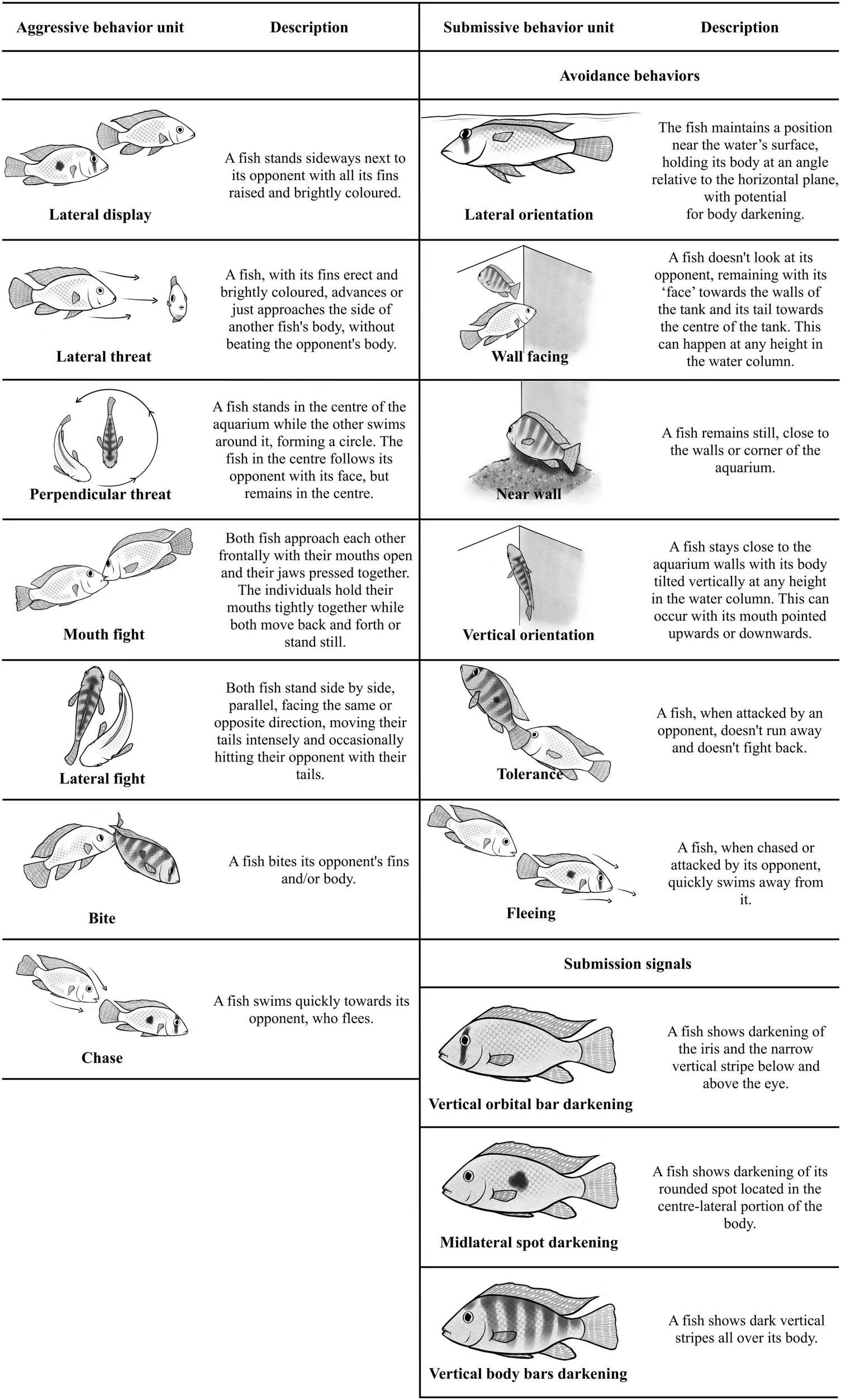
Ethogram of agonistic interactions of *Geophagus iporangensis*.

As *G. iporangensis* is a species that has not been widely studied in terms of animal behavior, before starting the tactile stimulation experiments, a study was carried out to characterize their agonistic behavior in both dominant and subordinate fish. In this study, the fish without sex identification were paired (*n* = 6 pairs, standard length = 9.02 ± 0.23 cm; weight = 24.06 ± 2.05 g; mean ± SE), and fights were recorded for 30 min. Based on these fights, an illustrated ethogram of the agonistic repertoire of *G. iporangensis* was developed to quantify subsequent behavioral analyses (Figure 2).

### 2.5 Appeasement behavior protocol

**FIGURE 3:**
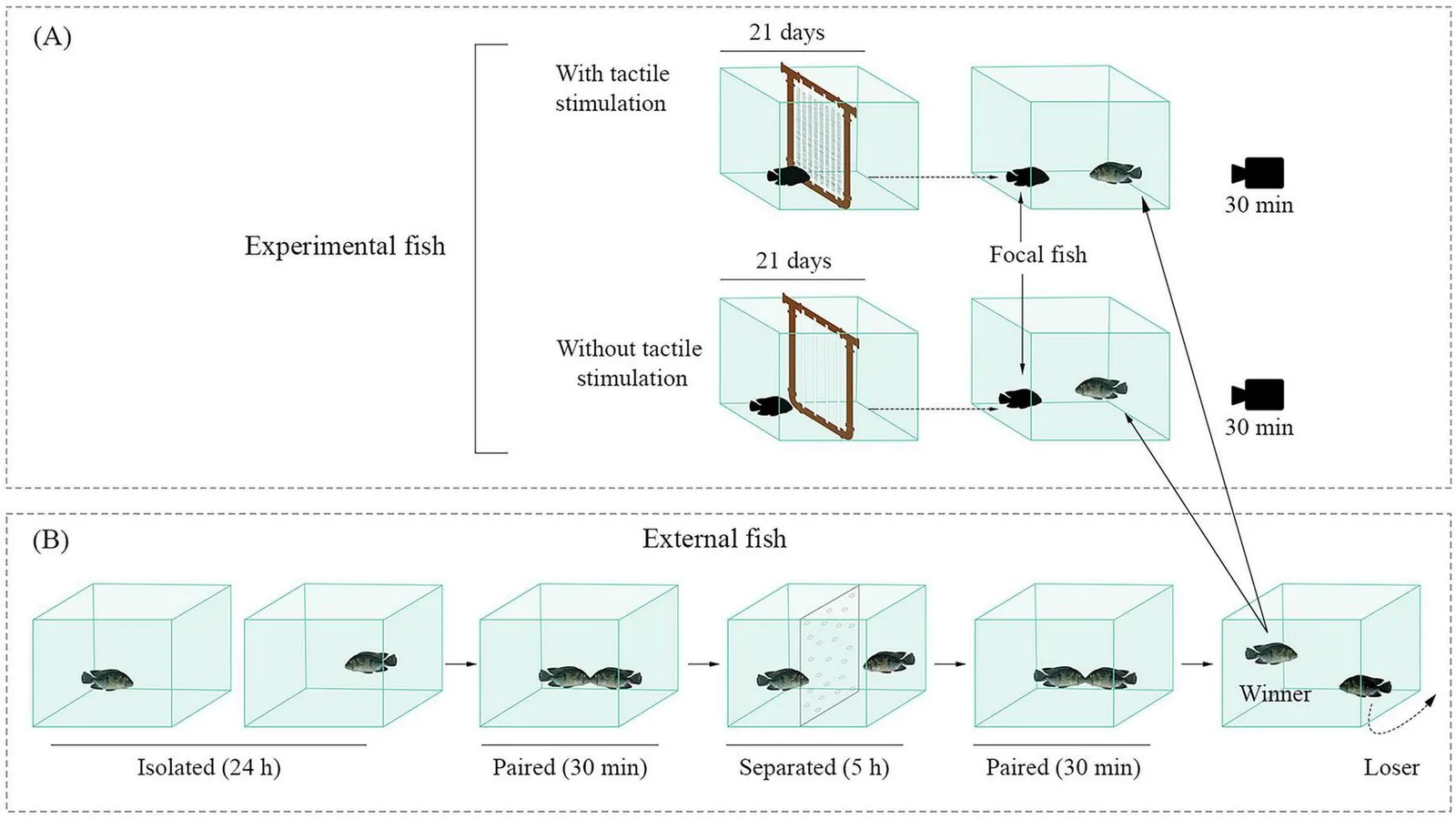
Experimental protocol for testing the effect of tactile stimulation (TS) on appeasement behavior. **(A)** Individual *G. iporangensis* were submitted to treatments with or without TS for 21 days (*N* = 13 individuals each treatment). Then, they were paired in a neutral aquarium with an external fish that had previously experienced dominance. **(B)** To establish the social rank of the external fish, individuals of similar size were isolated for 24 h, then allowed to interact for 30 min. Afterward, they were separated by a perforated transparent partition within the same aquarium. Five hours later, the partition was removed, and the fish interacted again to confirm their social ranks. The dominant (winner) fish was considered the one that attacked, was not attacked and chased the other fish that fled. On the other hand, the fish that fled from attacks or chases, and did not fight back when attacked, was considered the subordinate (loser). The loser was then removed from the aquarium. The following day, an experimental fish (with or without TS) was introduced into the dominant fish’s aquarium. The interactions were video recorded for 30 min to quantify appeasement behavior, considering the fish from treatments with or without TS as the focal one.

Fish from TS or without TS treatments were paired with a fish not involved in the experiment (external fish) with previous experience of dominance, as described in Figure 3. This strategy was used to favor the rapid resolution of the hierarchy and the emergence of submissive behaviors, also allowing the control of the previous social rank experience of the opponent. Besides dominance experience, the external animal was slightly bigger than the focal one (0.53 ± 0.11 cm and 5.68 ± 1.40 g larger than the focal fish; mean ± SE) and was also the resident in the aquarium, which helped to maintain the social rank (Wazlavek and Figler, 1989).

Individual recognition during fights was done by marking the dominant animals with colored elastomer (VIE tags) under the body scales. All pairs were video-recorded by cameras positioned on tripods in front of the aquariums. The cameras were set up just before the agonistic interaction began and removed once it was over. Initially, we tested 14 replicates in each group (with and without TS). However, in one pair of each treatment, the fish with prior dominance did not establish dominance over the focal fish, so that the social rank was inconclusive. Therefore, we removed these replicates and worked only with the remaining 13 replicates in each group, where all focal fish were clearly subordinate.

Submissive behaviors in cichlids are characterized as states, as they involve prolonged changes in body coloration and posture. Therefore, we used focal sampling with instantaneous recording (Bateson and Martin, 2021) to quantify these behaviors. The experimental fish were the focal one. Aggressive interactions were recorded for 30 min, with behaviors sampled every 15 s, totaling 120 intervals. For each interval, a “1” was assigned if a behavior occurred within the 15 s, and “0” if it did not.

For each of the nine behavioral units, a relative frequency was calculated by dividing the number of occurrences by the 120 total intervals (Supplementary material – Supplementary Table 1); this frequency served as the behavior’s intensity score. Following Reddon et al. (2021), we grouped these scores into two categories: Submission Signals (vertical orbital bar, midlateral spot, and vertical body bar darkening) and Avoidance Behaviors (vertical orientation, lateral orientation, near wall, wall facing, tolerance, and fleeing). The score for each category was the mean of its constituent unit scores. The Total Appeasement score was defined as the mean of all nine units.

Because submissive units can occur simultaneously and intensify as a fight escalates, for instance, an individual may display “vertical orientation” concurrently with “vertical body bar darkening” (Figure 2); we also investigated the intensity of the Total Appeasement over time. We calculated an aggregated score for each 5-min block (each block comprising 20 sampling intervals, and a total of 6 blocks) by summing the occurrences of all nine behavioral units and dividing the total by 20. This score represents the average number of simultaneous submissive behavioral units displayed per interval. With nine behavioral units possible, scores could range from 0.0 to 9.0 per interval.

### 2.6 Stress indicators

Stress was inferred by plasma cortisol levels (the main indicator of HPI axis activation in fish) as well as by length gain, weight gain, and specific growth rate (SGR), the latter indicating long-term stress in fish (Sopinka et al., 2016). Immediately after the fight against the dominant fish ended, the animals were anesthetized with Benzocaine (0.09 g/L) and blood was collected by tail vein puncture using hypodermic needles and heparinized syringes (15 μL of blood was collected from each fish). All blood samples were collected between 10 and 11 a.m. to avoid possible circadian variation. The whole procedure lasts less than 2 min to avoid the manipulation effect on cortisol levels (e.g., Pottinger, 2008). The blood was then centrifuged at 3,000 rpm for 10 min. The plasma was separated and frozen at −20°C for later cortisol assay. This analysis was carried out using ELISA (Enzyme Linked Immunosorbent Assay) commercial kits (Tecan IBL Immunobiological Laboratories, Hamburg, Germany; RE52061), following the supplier’s protocol. The analysis was performed in duplicate on all fish in the experiment, without sample dilution. The mean intra-assay coefficient of variation was 6.77%. All samples were analyzed on a single ELISA plate; therefore, there was no inter-plate variability.

The fish were weighed and measured at the beginning and end of the experiment. The length and weight gain were then calculated by subtracting the initial from the final measurements. SGR was calculated as SGR = {[ln(final weight) − ln(initial weight)]/time} × 100 (Lugert et al., 2016), being the time equal 21 days.

### 2.7 Statistics

Data were analyzed using R (R Core Team, 2024), version 4.3.3. Normality and homogeneity of the data were tested using the Shapiro-Wilk and Levene tests, respectively. When data met these assumptions, parametric tests were used. When assumptions were violated, generalized linear models (GLMs) were fitted to account for non-normality. Statistical significance was defined as *p* ≤ 0.05.

To analyze the effect of treatment and days on the number of crossings through the apparatus over the 21 days of the experiment, we fitted a generalized linear mixed model (GLMM) with normal distribution (“glmmTMB” package). The model included “treatment” (with and without tactile stimulation) and “day” (categorical variable) as fixed effects, and the interaction between “treatment” and “day.” The random effect was modeled to account for individual variation in the replicates.

To compare the relative frequencies of each appeasement behavior between groups, we used a GLM with ordered beta distribution (“glmmTMB” package). To compare the appeasement scores of Avoidance Behaviors and Submission Signals between the two groups, we used a Student’s *T*-test for independent samples. To compare the Total Appeasement score between the two groups, we used a generalized linear model (GLM) with beta distribution (“glmmTMB” package).

For the appeasement score over time in the fight, we fitted a GLMM with a normal distribution to analyze the effect of the treatment, interval and their interaction on the score (“glmmTMB” package). The model included “treatment” (with and without tactile stimulation) and “intervals” (categorical variable) as fixed effects, and the interaction between “treatment” and “intervals.” The random effect was modeled to account for individual variation in the replicates. Tukey’s HSD (’emmeans’ package) was used to compare means between and within groups.

Growth indicators were compared using Student’s *T*-test for independent samples. The same was done for cortisol levels.

## 3 Results

**FIGURE 4:**
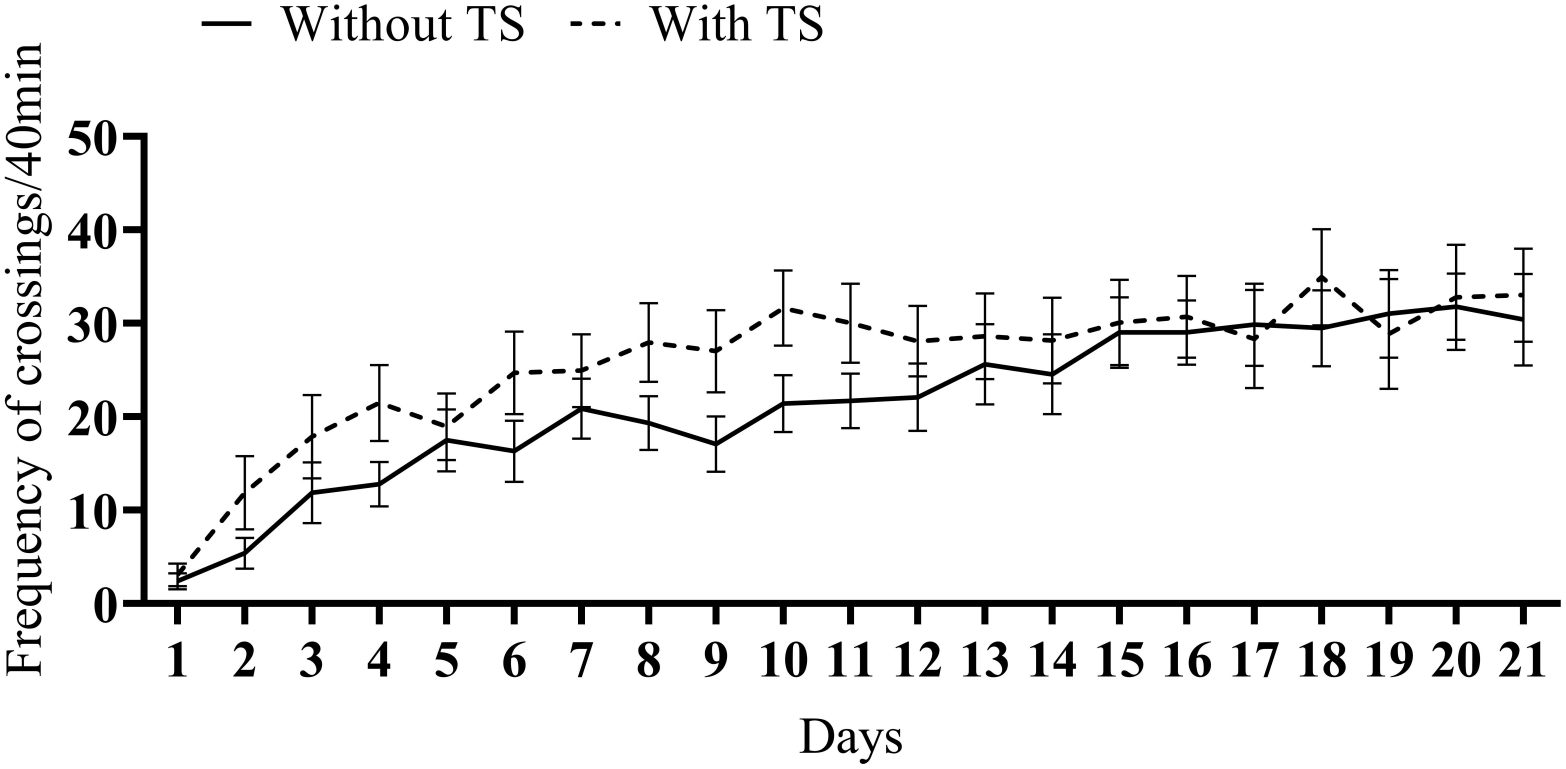
Apparatus efficiency. Frequency of individuals crossing in the groups with and without tactile stimulation over time. There were no significant differences between groups (GLMM with normal distribution), but crossing frequency increased significantly over time (χ^2^ = 362.06; *p* < 0.0001). Data are mean ± SE.

The effect of treatment on the frequency of crossings did not change according to the day (interaction: χ^2^_(20)_ = 20.76; *p* = 0.41). The animals increased their crossing frequency over the days (main effect of day: χ^2^_(20)_ = 362.06; *p* < 0.0001), while the animals similarly crossed the apparatus between groups (main effect of treatment: χ^2^_(1)_ = 1.20; *p* = 0.27) (Figure 4).

Except for the “vertical orientation,” which was marginally higher for the TS group (χ^2^_(1)_ = 3.51; *p* = 0.06), no significant differences were found between groups in the relative frequencies of appeasement behaviors, the grouped scores (Avoidance Behaviors and Submission Signals), or the Total Appeasement score (Table 1).

**TABLE 1:** Appeasement behavior scores and Total Appeasement score in groups with and without stimulation (control).

Behaviors	Score without TS	Score with TS	Test value	*P*-value
Avoidance behaviors	0.17 ± 0.02	0.19 ± 0.03	t (24) = 0.64	*p* = 0.53
• Lateral orientation	0.08 ± 0.05	0.09 ± 0.03	χ^2^ (1) < 0.0001	*p* = 0.99
• Wall facing	0.31 ± 0.08	0.20 ± 0.06	χ^2^ (1) = 1.00	*p* = 0.32
• Near wall	0.31 ± 0.08	0.46 ± 0.07	χ^2^ (1) = 3.28	*p* = 0.07
• Vertical orientation	0.18 ± 0.05	0.28 ± 0.07	χ^2^ (1) = 3.51	*p* = 0.06
• Tolerance	0.06 ± 0.03	0.03 ± 0.01	χ^2^ (1) = 0.003	*p* = 0.96
• Fleeing	0.08 ± 0.02	0.09 ± 0.02	χ^2^ (1) = 0.24	*p* = 0.62
Submission signals	0.34 ± 0.06	0.44 ± 0.07	t (24) = 1.13	*p* = 0.27
• Vertical orbital bar darkening	0.37 ± 0.10	0.42 ± 0.09	χ^2^ (1) = 0.04	*p* = 0.85
• Midlateral spot darkening	0.19 ± 0.05	0.32 ± 0.07	χ^2^ (1) = 1.81	*p* = 0.18
• Vertical body bars darkening	0.47 ± 0.08	0.59 ± 0.09	χ^2^ (1) = 1.01	*p* = 0.31
Total Appeasement	0.23 ± 0.03	0.27 ± 0.04	χ^2^ (1) = 0.67	*p* = 0.41

Data were analyzed using a generalized linear model (GLM) with ordered beta distribution for individual behaviors and beta distribution for the total score. Grouped behavior scores were compared between groups using an unpaired *t*-test. Data are mean ± SE.

**FIGURE 5:**
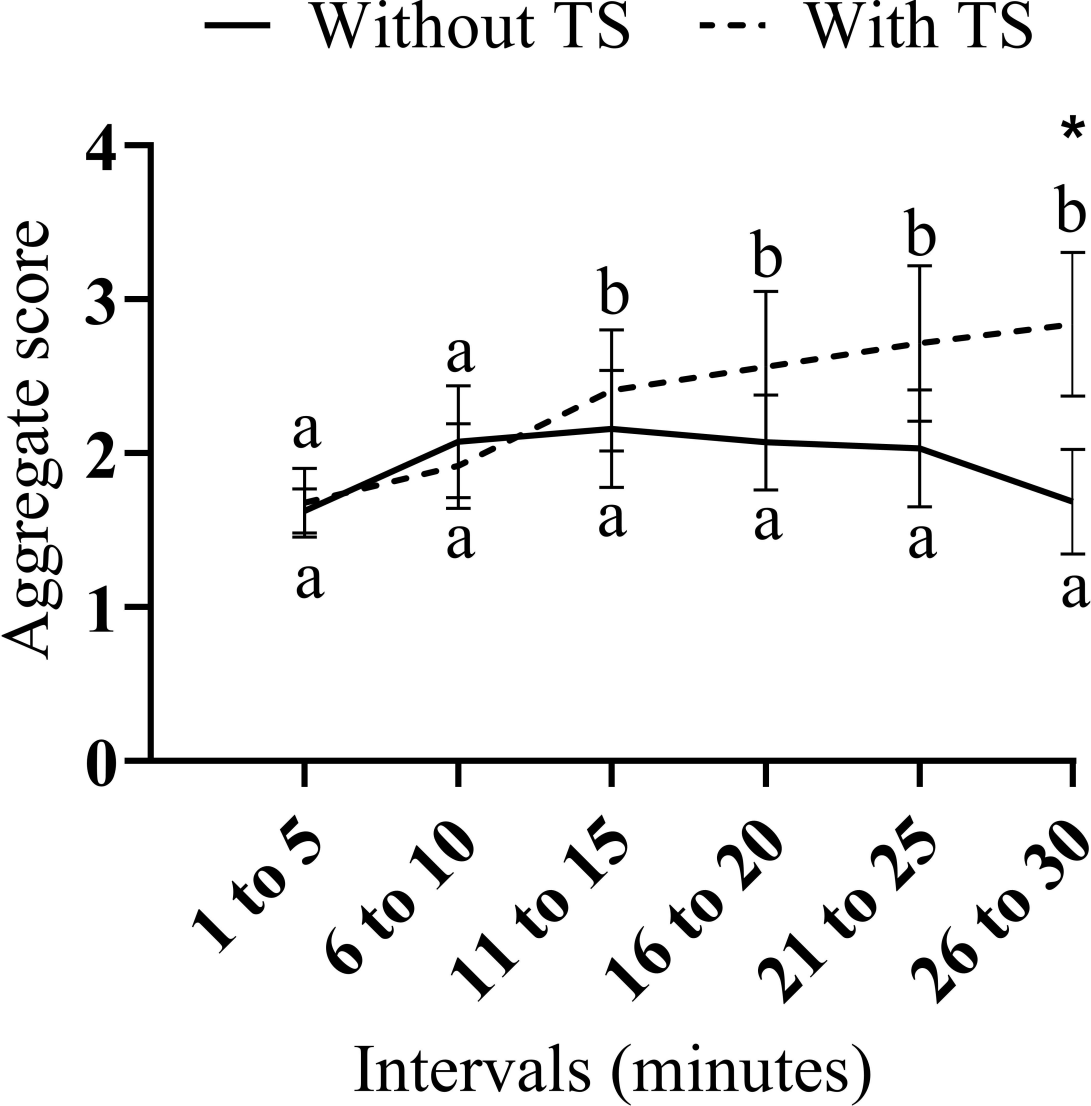
Appeasement score over 5 min interval. Data analyzed in blocks of 5 min over the course of the fight. In this case, the score was aggregated in each 5 min time interval, not being summed up over further intervals; asterisks indicate a significant difference in scores between groups (GLMM with normal distribution, and Bonferroni *post hoc* test), with the tactile stimulation group showing higher appeasement scores from minute 26. Tukey HSD was used to compare intervals within treatments, with differences from the first interval indicated by different letters. Data are mean ± SE. *p < 0.05.

Analyzing the intensity of appeasement over time, we found that the effect of treatment on the score changed according to the interval (χ^2^_(5)_ = 17.01; *p* = 0.005), with a difference in the interval from 26 to 30 min (Tukey; *p* = 0.01) for the group with TS (Figure 5). There was also an effect of time intervals on the score (χ^2^_(5)_ = 25.17; *p* = 0.0001), while the accumulated scores were similar between groups overall (GLMM with normal distribution; χ^2^_(1)_ = 1.01; *p* = 0.32). Differences were also found within groups when comparing the first interval with the following ones (Tukey; TS:1*vs*2 *p* = 0.81, 1*vs*3 *p* = 0.03, 1*vs*4 *p* = 0.003, 1*vs*5 *p* = 0.0003, 1*vs*6 *p* = 0.0001. Without TS: 1*vs*2 *p* = 0.50, 1*vs*3 *p* = 0.37, 1*vs*4 *p* = 0.59, 1*vs*5 *p* = 0.62, 1*vs*6 *p* = 0.99).

No significant differences were found between groups for length gain (mean ± SE; with TS 0.07 ± 0.09 cm; without TS 0.06 ± 0.04 cm; t _(24)_ = 0.08; *p* = 0.94), weight gain (mean ± SE; with TS −0.04 ± 0.44 g; without TS 0.76 ± 0.35 g; t _(24)_ = −1.45; *p* = 0.16), SGR (mean ± SE; with TS 0.05 ± 0.12 %; without TS 0.16 ± 0.08 %; t _(24)_ = −0.80; *p* = 0.43) and cortisol levels (mean ± SE; with TS 345.26 ± 38.04 cm; without TS 296.33 ± 44.54; t _(24)_ = 0.84; *p* = 0.41). The raw dataset resulting from this original research is available in the Supplementary Dataset 2.

## 4 Discussion

In this study, we observed that the artificial apparatus was effective in providing tactile stimulation, as individuals spontaneously crossed it throughout the treatment period. We found that individuals who received tactile stimulation had a higher aggregated appeasement score at the end of the fight, therefore supporting our hypothesis that tactile stimulation intensifies appeasement behavior in fish. On the other hand, no differences in stress levels were found between the two groups. We observed that the frequency that fish crossed the apparatus increased over the days in both groups. This indicates that the apparatus was not aversive to the fish, as the number of crossings did not decrease over time, but rather increased and then remained constant. It also indicates that, like Nile tilapia, *G. iporangensis* needs some time to explore the environment in the presence of the apparatus (Bolognesi et al., 2019; Gauy et al., 2022). In fact, fish usually need time to adjust to novel environmental stimuli (Hamilton, 2018), which appears to have occurred in this study. The silicone bristles of the TS apparatus, despite being soft and transparent, can act as a mechanical and visual barrier for the fish, usually resulting in fewer crossings when compared to the control treatment (Gauy et al., 2022). However, for *G. iporangensis*, the bristles did not interfere with movement through the environment, as animals in the treatment group crossed through the apparatus similarly to those in the control group. Therefore, the apparatus was an efficient environmental enrichment structure and suitable for testing several hypotheses regarding tactile stimulation in fish.

Social communication often involves combining signals from the same or different sensorial modalities (Partan and Marler, 1999), which is also true for cichlids whose communicate by chemical (Keller-Costa et al., 2015; Gonçalves-de-Freitas et al., 2008; Gauy et al., 2018; Giaquinto and Volpato, 1997), acoustic (Nelissen, 1978; Amorim et al., 2019), and visual signals (John et al., 2021; Sefc et al., 2014). Previous studies have shown that visual signals such as body coloration and postural changes are sufficient to reduce the aggressiveness of the opponent (Ruberto et al., 2020; Miyai et al., 2011). If a fish changes its coloration and at the same time adopts a submissive posture, the combination of these two visual signals will convey its lower aggressive motivation to other animals. This reinforcement will increase the likelihood of being perceived by the receiver (Partan and Marler, 1999). Given the importance of visual signals in cichlid communication, we used scores to analyze aggressive behavior and quantify the intensity of this communication. It is also possible that other sensory signals were released alongside the visual signals, accelerating the establishment of social hierarchy (e.g., Gonçalves-de-Freitas et al., 2008; Giaquinto and Volpato, 1997). However, it was not possible to quantify chemicals or sounds in this study. Nevertheless, we consider that visual signals were a reliable indicator of appeasement behavior in *G. iporangensis*.

We found no effect of TS treatment on any of the submissive behavior units, either when considered individually or when grouped according to Reddon et al. (2021). However, the “vertical orientation” showed a marginally significant difference, with those with TS exhibiting this posture more often than those without TS, suggesting that this posture may serve as a stronger signal of submission. In fact, a similar posture appears to be used to signal submission in *Neolamprologus pulcher*, the daffodil cichlid (Ruberto et al., 2020). In this species, when the posture was exhibited by the subordinate signalers in response to aggression, the other fish took longer to behave aggressively toward them again (Ruberto et al., 2020). *N. pulcher* is a cooperative breeding species endemic to the Lake Tanganyika (Balshine-Earn et al., 1998) that forms social hierarchy groups (Wong and Balshine, 2010). Unlike the hierarchical dynamics observed in *G. iporangensis*, subordinate daffodil cichlids renounce their own reproductive needs to help dominant breeders raise their offspring (Wong and Balshine, 2010). Despite their distinct social structures, the vertical position appears to play a meaningful role in signaling submission in both species. The clear effect observed in daffodil cichlids (Ruberto et al., 2020) is consistent with the demands of their cooperative social system. Because subordinates must bear direct reproductive costs, renouncing their ability to reproduce independently in exchange for territorial protection and the prospect of inheriting the natal territory (Balshine-Earn et al., 1998), effectively signaling submission has significant fitness implications, making reliable communication of submissive status particularly adaptive. Conversely, while *G. iporangensis* does not exhibit such cooperative breeding, the marginally significant difference in vertical posture observed in the present study suggests that this signal may retain communicative relevance even outside cooperative contexts, pointing to a conserved postural signaling function across cichlid social hierarchies. Nevertheless, further research is necessary to understand the precise role of such postures in the social dynamics of *G. iporangensis* and the context specificity of its submissive signals.

Although no significant differences were found between behavioral units, a significant result was found for the aggregated score over time. When considering the temporal progression of the fight between treatments, we found that fish from the TS group showed increased appeasement score in the final moments, indicating a delayed effect of the stimulation on appeasement behavior. But when considering within groups, scores in the TS increased significantly after 10 min, while they did not increase in the control group, therefore showing a clear effect of TS on the appeasement behavior. This result suggests that tactile stimulation may influence the dynamics of rank settlement over time. Thus, assessing submission through a cross-sectional approach, i.e., evaluating appeasement behavior in isolation without considering temporal progression, does not seem appropriate in the context of competition for social position. Indeed, submission is a dynamic process in which behaviors overlap throughout the duration of the fight, with different visual signals simultaneously influencing the intensity of the individual response (Partan and Marler, 1999). In our study, however, the aggressive behavior exhibited by the dominant was not quantified. It could be that the increased appeasement behavior at the end of the fight was due to increased aggression by the dominant in the TS group (although differences in the dominants’ behavior from the TS or control group were not expected, as they were not subjected to the experimental apparatus). It is known that appeasement behavior may inhibit the dominant’s aggression and facilitate the end of conflicts without escalation, with the submission signals acting as an adaptive strategy for subordinates to establish and maintain their position in the hierarchy, while avoiding prolonged aggressive interactions with dominants (Reddon et al., 2019; Ruberto et al., 2020). In the cooperative breeding species *N. pulcher*, for example, submissive behavior acts as a pre-emptive strategy to prevent dominant attacks, rather than a proportional response to ongoing aggression (Bergmüller and Taborsky, 2005), also promoting hierarchy stability by increasing dominant tolerance (Hick et al., 2014). Whether a pre-emptive appeasement strategy is conserved across cichlid species, and whether dominants’ aggression contributed to the observed pattern in this study, are questions that future studies should address by simultaneously tracking submissive and aggressive behaviors throughout interaction.

Tactile stimulation, also referred to as “touch,” is known to have positive effects in mammals, by reducing stress and depression (Freitas et al., 2015), and maintaining social group cohesion (Dunbar, 1991: Lehmann et al., 2007). Artificial TS has also been shown to have positive effects, such as reducing anxiety-like behaviors in rats (Freitas et al., 2015), reducing aggression in feedlot steers (Park et al., 2020) and soothing in dog puppies (Gazzano et al., 2008). The general mechanism by which TS has positive effects on mammals is related to specific skin mechanoreceptors that stimulate C-tactile fibers that, when activated, release neurotransmitters such as dopamine, serotonin, oxytocin (Adams and Moghaddam, 2000; Walker et al., 2017), and endocannabinoids (Jones et al., 2025) in different brain areas. In fish, the mechanosensory cells are distributed along the lateral line, fins, and other regions of the skin (Butler and Maruska, 2016b). Similar to mammals, mechanostimulation appears to be associated with serotonergic pathways (Devitsina and Lapshin, 2020). In fact, Abreu et al. (2018), found higher levels of the main oxidative metabolite of serotonin, 5-HIAA in the forebrain and midbrain of client fish exposed to tactile stimulation provided by cleaner fish. The activation of serotonergic pathways is also associated with aggressive behavior and social rank in fish. In salmonids, high levels of 5-HT activity are associated with low levels of aggression and submissive behavior, while low levels of 5-HT activity are associated with high levels of aggression and dominance (Lillesaar, 2011). Although we have not dug into monoaminergic mechanisms, it is plausible to assume that tactile stimulation would also affect fish aggressiveness by stimulating serotonergic pathways.

We analyzed weight gain, length gain and SGR between groups with and without tactile stimulation. These growth indicators are important as malnutrition in fish is often associated with an increase in aggressiveness (Chandararathna et al., 2021), which could affect not only the welfare of these individuals but also the results of our research. However, there was no effect of tactile stimulation on the growth of *G. iporangensis*. Our results contrast with those found in Nile tilapia (Gauy et al., 2022), where the long period of tactile stimulation increased SGR and weight gain. A possible explanation is that *G. iporangensis* may grow more slowly compared to Nile tilapia, as the later have been artificially selected for faster growth rates (de Oliveira et al., 2016). In fact, Nile tilapia appears to have a faster growth curve (Carvalho et al., 2021) than *G. iporangensis* (Fontoura and Santos, 2002), indicating that 21 days may not be enough time to show significant growth in these species. Thus, while not promoting growth, there was no reduction either, suggesting that the fish exposed to TS did not experience distress (Wendelaar-Bonga, 1997).

Plasma cortisol levels did not differ between experimental groups. Tactile stimulation has already been shown to be effective in reducing stress in *Ctenochaetus striatus*, a tropical fish species that naturally receives tactile stimulation from a cleaner fish, *Labroides dimidiatus*, on coral reefs (Soares et al., 2011). However, the conditions observed in our study were different from those tested for *C. striatus*, as the TS protocol varied between the two studies (we used a tactile apparatus to apply the TS, whereas the previous study employed a mechanical device). Thus, this similarity in cortisol levels may be related to the natural behavior of cichlids, which is different from that of species that exhibit client-cleaner interactions. However, our results are similar to those found in Nile tilapia, where the introduction to the tactile stimulation apparatus also did not affect cortisol (Bolognesi et al., 2019; Gauy et al., 2022). The absence of this effect in our study may also be associated with the moment of blood sampling, which was performed immediately after the fights, reflecting the acute state of fighting and submission (Devries et al., 2003). Some studies show an increase in cortisol levels in subordinate fish, such as the neotropical cichlid *Aequidens pulcher*, whose high levels of submission were associated with high levels of cortisol (Munro and Pitcher, 1985). Thus, it is possible that, even with a more intense appeasement behavior, the elevated cortisol levels in *G. iporangensis* were due to acute effects of fighting, which may have covered up the effect of TS.

Since there is no external sexual dimorphism for *G. iporangensis*, both sexes were analyzed. Therefore, we acknowledge the potential for differences in behavior attributed to sex as a limitation of the present work. For some fish species, sex has a strong effect on their behavior. In zebrafish, for example, males and females differ in the intensity of aggression when exposed to unpredictable chronic stress (Rambo et al., 2017). On the other hand, both males and females of Neotropical cichlids actively participate in parental care and display aggressive behaviors to defend offspring and territory, so both engage in aggressive interactions (Holder et al., 1991; Teresa and Gonçalves-de-Freitas, 2011). For example, in *Cichlasoma dimerus*, there is no difference in aggressive and submissive behavior between males and females (Scaia et al., 2023), and females display aggressive behavior naturally (Scaia et al., 2018, 2020). Complementarily, aggressive motivation does not differ between males and females of *Amatitlania nigrofasciata*, indicating that both value the defended resource proportionally, despite differences in their respective aggressive repertoires (Arnott and Elwood, 2009). Even though there is no specific data for *G. iporangensis* in the literature, the aggressiveness between males and females may be similar to that found in other biparental Neotropical cichlid species, with both sexes having similar levels of aggressive interactions.

The effect of TS on cichlids observed here suggests the existence of a natural sensory system that responds to touch. The presence of tactile interactions in the natural repertoire of cichlids is little explored in the literature. However, mechanosensory signals elicited by hydrodynamic stimulation and detected by the lateral line are considered important in the context of communication during social interactions (Butler and Maruska, 2015, 2016a). Furthermore, this mechanosensory system provides relevant information to the decision-making centers of the brain during territorial interactions in cichlids (Butler and Maruska, 2016b). Thus, tactile stimulation may be part of their behavioral repertoire, e.g., during courtship or in other interactions with conspecifics. In this context, our data suggest that TS may be a basic form of social touch that regulates social behavior in vertebrates by triggering appeasement behavior.

## Data Availability

The original contributions presented in this study are included in this article/Supplementary material, further inquiries can be directed to the corresponding author.

## References

[B1] Abreu M. Messias J. Thörnqvist P. Winberg S. Soares M. (2018). Monoaminergic levels at the forebrain and diencephalon signal for the occurrence of mutualistic and conspecific engagement in client reef fish. *Sci. Rep.* 8:7346. 10.1038/s41598-018-25513-6 29743658 PMC5943261

[B2] Adams B. Moghaddam B. (2000). Tactile stimulation activates dopamine release in the lateral septum. *Brain Res.* 858 177–180. 10.1016/S0006-8993(99)02473-7 10700612

[B3] Amorim M. Fonseca P. Mathevon N. Beauchaud M. (2019). Assessment of fighting ability in the vocal cichlid Metriaclima zebra in face of incongruent audiovisual information. *Biol. Open* 8:bio043356. 10.1242/bio.043356 31852657 PMC6955207

[B4] Arechavala-Lopez P. Cabrera-Álvarez M. J. Maia C. M. Saraiva J. L. (2022). Environmental enrichment in fish aquaculture: A review of fundamental and practical aspects. *Rev. Aquac.* 704–728. 10.1111/raq.12620

[B5] Arnott G. Elwood R. (2009). Gender differences in aggressive behaviour in convict cichlids. *Anim. Behav.* 78 1221–1227. 10.1016/j.anbehav.2009.08.005

[B6] Aureli F. Cords M. Van Schaik C. (2002). Conflict resolution following aggression in gregarious animals: A predictive framework. *Anim. Behav.* 64 325–343. 10.1006/anbe.2002.3071

[B7] Balshine S. Wong M. Reddon A. (2017). Social motivation and conflict resolution tactics as potential building blocks of sociality in cichlid fishes. *Behav. Process.* 141 152–160. 10.1016/j.beproc.2017.01.001 28057516

[B8] Balshine-Earn S. Neat F. Reid H. Taborsky M. (1998). Paying to stay or paying to breed? Field evidence for direct benefits of helping behavior in a cooperatively breeding fish. *Behav. Ecol.* 9 432–438. 10.1093/beheco/9.5.432

[B9] Barata E. Hubbard P. Almeida O. Miranda A. Canário A. (2007). Male urine signals social rank in the Mozambique tilapia (Oreochromis mossambicus). *BMC Biol.* 5:54. 10.1186/1741-7007-5-54 18076759 PMC2222621

[B10] Barley A. Coleman R. (2010). Habitat structure directly affects aggression in convict cichlids Archocentrus nigrofasciatus. *Curr. Zool.* 56 52–56. 10.1093/czoolo/56.1.52

[B11] Barlow G. (2000). *The Cichlid Fishes: Nature’s Grand Experiment in Evolution*, 1st Edn. New York, NY: Basic Books.

[B12] Bateson M. Martin P. (2021). *Measuring Behaviour: an Introductory Guide*, 4th Edn. Cambridge, MA: Cambridge University Press, 10.1017/9781108776462

[B13] Bergmüller R. Taborsky M. (2005). Experimental manipulation of helping in a cooperative breeder: helpers ‘pay to stay’by pre-emptive appeasement. *Anim. Behav.* 69 19–28. 10.1016/j.anbehav.2004.05.009

[B14] Bolognesi M. Gauy A. Gonçalves-de-Freitas E. (2019). Tactile stimulation reduces aggressiveness but does not lower stress in a territorial fish. *Sci. Rep.* 9:40. 10.1038/s41598-018-36876-1 30631114 PMC6328608

[B15] Bshary R. Côté I. (2008). New perspectives on marine cleaning mutualism. *Fish Behav.* 563–592.

[B16] Butler J. Maruska K. (2015). The mechanosensory lateral line is used to assess opponents and mediate aggressive behaviors during territorial interactions in an African cichlid fish. *J. Exp. Biol.* 218 3284–3294. 10.1242/jeb.125948 26491195

[B17] Butler J. Maruska K. (2016a). Mechanosensory signaling as a potential mode of communication during social interactions in fishes. *J. Exp. Biol.* 219 2781–2789. 10.1242/jeb.133801 27655819

[B18] Butler J. Maruska K. (2016b). The mechanosensory lateral line system mediates activation of socially-relevant brain regions during territorial interactions. *Front. Behav. Neurosci.* 10:93. 10.3389/fnbeh.2016.00093 27242462 PMC4865491

[B19] Carvalho J. C. Filho R. A. C. Oliveira C. A. L. Ribeiro R. P. Seraphim G. N. Silva A. L. N. et al.. (2021). Growth curve of Nile tilapia from different families of the AquaAmérica variety. *Braz. J. Biol.* 82:e243534. 10.1590/1519-6984.243534 34133573

[B20] Cetra M. Garibaldi L. Teresa F. Peressin A. Cruz B. de Mello B. et al.. (2022). Taxonomic and functional diversity patterns of stream fish assemblages from Brazilian Atlantic Rain Forest. *Fisheries Manag. Ecol.* 29 911–920. 10.1111/fme.12592

[B21] Chandararathna U. Iversen M. Korsnes K. Sørensen M. Vatsos I. (2021). Animal welfare issues in capture-based aquaculture. *Animals* 11:956. 10.3390/ani11040956 33808163 PMC8065896

[B22] Copeland D. Levay B. Sivaraman B. Beebe-Fugloni C. Earley R. (2011). Metabolic costs of fighting are driven by contest performance in male convict cichlid fish. *Anim. Behav.* 82 271–280. 10.1016/j.anbehav.2011.05.001

[B23] de Oliveira C. Ribeiro R. Yoshida G. Kunita N. Rizzato G. de Oliveira S. et al.. (2016). Correlated changes in body shape after five generations of selection to improve growth rate in a breeding program for Nile tilapia Oreochromis niloticus in Brazil. *J. Appl. Genet.* 57 487–493. 10.1007/s13353-016-0338-5 26810351

[B24] Devitsina G. Lapshin D. (2020). A noninvasive electrophysiological investigation of tactile sensitivity in cyprinid fish (Cyprinidae). *J. Evol. Biochem. Phys.* 56 338–347. 10.1134/S0022093020040055

[B25] Devries A. Glasper E. Detillion C. (2003). Social modulation of stress responses. *Physiol. Behav.* 79 399–407. 10.1016/S0031-9384(03)00152-5 12954434

[B26] Dunbar R. (1991). Functional significance of social grooming in primates. *Folia primatol.* 57 121–131. 10.1159/000156574

[B27] Elwood R. Arnott G. (2012). Understanding how animals fight with Lloyd Morgan’s canon. *Anim. Behav.* 84 1095–1102. 10.1016/j.anbehav.2012.08.035

[B28] Firnkes A. Bartels A. Bidoli E. Erhard M. (2017). Appeasement signals used by dogs during dog–human communication. *J. Vet. Behav.* 19 35–44. 10.1016/j.jveb.2016.12.012

[B29] Fontoura N. Santos G. (2002). Idade e crescimento de Geophagus brasiliensis (quoy & gaimard, 1824). no açude Águas Belas, Viamão, Rio Grande do Sul (Teleostei: Cichlidae). *Comum. Mus. Ciênc. Tecnol. PUCRS* 15 215–225. Portuguese.

[B30] Freitas D. Antoniazzi C. Segat H. Metz V. Vey L. Barcelos R. et al.. (2015). Neonatal tactile stimulation decreases depression-like and anxiety-like behaviors and potentiates sertraline action in young rats. *Int. J. Dev. Neurosci.* 47 192–197. 10.1016/j.ijdevneu.2015.09.010 26449401

[B31] Frommen J. (2020). Aggressive communication in aquatic environments. *Funct. Ecol.* 34 364–380. 10.1111/1365-2435.13482

[B32] Gauy A. Bolognesi M. Gonçalves-de-Freitas E. (2022). Long-term body tactile stimulation reduces aggression and improves productive performance in Nile tilapia groups. *Sci. Rep.* 12:20239. 10.1038/s41598-022-24696-3 36424460 PMC9691712

[B33] Gauy A. Bolognesi M. Gonçalves-de-Freitas E. (2023). Body tactile stimulation reduces the effects of high stocking density on the welfare of nile tilapia (Oreochromis niloticus). *Fishes* 8:320. 10.3390/fishes8060320

[B34] Gauy A. Bolognesi M. Martins G. Gonçalves-de-Freitas E. (2021). Preference and motivation tests for body tactile stimulation in fish. *Animals* 11:2042. 10.3390/ani11072042 34359170 PMC8300383

[B35] Gauy A. Boscolo C. Gonçalves-de-Freitas E. (2018). Less water renewal reduces effects on social aggression of the cichlid Pterophyllum scalare. *Appl. Anim. Behav. Sci.* 198 121–126. 10.1016/j.applanim.2017.10.003

[B36] Gazzano A. Mariti C. Notari L. Sighieri C. McBride E. (2008). Effects of early gentling and early environment on emotional development of puppies. *Appl. Anim. Behav. Sci.* 110 294–304. 10.1016/j.applanim.2007.05.007

[B37] Giaquinto P. Volpato G. (1997). Chemical communication, aggression, and conspecific recognition in the fish Nile tilapia. *Physiol. Behav.* 62 1333–1338. 10.1016/S0031-9384(97)00347-8 9383122

[B38] Gonçalves-de-Freitas E. Bolognesi M. Gauy A. Brandão M. Giaquinto P. Fernandes-Castilho M. (2019). Social behavior and welfare in Nile tilapia. *Fishes* 4:23. 10.3390/fishes4020023

[B39] Gonçalves-de-Freitas E. Teresa F. Gomes F. Giaquinto P. (2008). Effect of water renewal on dominance hierarchy of juvenile Nile tilapia. *Appl. Anim. Behav. Sci.* 112 187–195. 10.1016/j.applanim.2007.07.002

[B40] Hamilton T. (2018). Object novelty and object location recognition memory in fish–Recent advances. *Handb. Behav. Neurosci.* 27 151–161. 10.1016/B978-0-12-812012-5.00009-4

[B41] Haseman J. D. (1911). An annotated catalog of the cichlid fishes collected by the expedition of the Carnegie Museum to central South America, 1907-10. *Ann. Carnegie Mus.* 7, 329–373. 10.5962/p.242803

[B42] Hick K. Reddon A. O’Conner C. Balshine S. (2014). Strategic and tactical fighting decisions in cichlid fishes with divergent social systems. *Behaviour* 151 47–71. 10.1163/1568539X-00003122

[B43] Holder J. Barlow G. Francis R. (1991). Differences in aggressiveness in the Midas cichlid fish (Cichlasoma citrinellum). in relation to sex, reproductive state and the individual. *Ethology* 88 297–306. 10.1111/j.1439-0310.1991.tb00284.x

[B44] John L. Rick I. Vitt S. Thünken T. (2021). Body coloration as a dynamic signal during intrasexual communication in a cichlid fish. *BMC Zool.* 6:9. 10.1186/s40850-021-00075-9 37170176 PMC10127425

[B45] Jones M. Haggarty C. Petrie G. Lunge A. Morrison I. Hill M. et al.. (2025). Endocannabinoid contributions to the perception of socially relevant, affective touch in humans. *Neuropsychopharmacol.* 50 849–855. 10.1038/s41386-025-02053-y 39843850 PMC11914470

[B46] Judge P. G. de Waal F. B. (1993). Conflict avoidance among rhesus monkeys: Coping with short-term crowding. *Anim. Behav.* 46 221–232. 10.1006/anbe.1993.1184

[B47] Kadry V. Barreto R. (2010). Environmental enrichment reduces aggression of pearl cichlid, Geophagus brasiliensis, during resident-intruder interactions. *Neotrop. Ichthyol.* 8 329–332. 10.1590/S1679-62252010000200011

[B48] Keller-Costa T. Canário A. Hubbard P. (2015). Chemical communication in cichlids: a mini-review. *Gen. Comp. Endocrinol.* 221 64–74. 10.1016/j.ygcen.2015.01.001 25622908

[B49] Lehmann J. Korstjens A. Dunbar R. (2007). Group size, grooming and social cohesion in primates. *Anim. Behav.* 74 1617–1629. 10.1016/j.anbehav.2006.10.025

[B50] Lillesaar C. (2011). The serotonergic system in fish. *J. Chem. Neuroanat.* 41 294–308. 10.1016/j.jchemneu.2011.05.009 21635948

[B51] Lopez-Fernandez H. Winemiller K. Montaña C. Honeycutt R. (2012). Diet-morphology correlations in the radiation of South American geophagine cichlids (Perciformes: Cichlidae: Cichlinae). *PLoS Ones* 7:e33997. 10.1371/journal.pone.0033997 22485154 PMC3317448

[B52] Lugert V. Thaller G. Tetens J. Schulz C. Krieter J. (2016). A review on fish growth calculation: Multiple functions in fish production and their specific application. *Rev. Aquac.* 8 30–42. 10.1111/raq.12071

[B53] Maximino C. Araujo-Silva H. Cacela-Rodrigues I. Luchiari A. Saraiva J. Soares M. (2025). The hedonic impact of cleaner–client fish interactions is mediated by the opioid system. *Proc. R. Soc. B* 292:20251532. 10.1098/rspb.2025.1532 40795973 PMC12343142

[B54] Miyai C. Sanches F. Costa T. Colpo K. Volpato G. Barreto R. (2011). The correlation between subordinate fish eye colour and received attacks: A negative social feedback mechanism for the reduction of aggression during the formation of dominance hierarchies. *Zoology* 114 335–339. 10.1016/j.zool.2011.07.001 21975142

[B55] Munro A. Pitcher T. (1985). Steroid hormones and agonistic behavior in a cichlid teleost. *Aequidens pulcher. Horm. Behav.* 19 353–371. 10.1016/0018-506X(85)90034-0 4085992

[B56] Nelissen M. (1978). Sound production by some tanganyikan cichlid fishes and a hypothesis for the evolution of the r communication mechanisnis. *Behaviour* 64 137–147. 10.1163/156853978X00477 629731

[B57] Oyakawa O. Menezes N. (2011). Checklist dos peixes de água doce do Estado de São Paulo. *Brasil. Biota Neotropica* 11 19–32. Portuguese. 10.1590/S1676-06032011000500002

[B58] Park R. Schubach K. Cooke R. Herring A. Jennings J. Daigle C. (2020). Impact of a cattle brush on feedlot steer behavior, productivity and stress physiology. *Appl. Anim. Behav. Sci.* 228:104995. 10.1016/j.applanim.2020.104995

[B59] Partan S. Marler P. (1999). Communication goes multimodal. *Science* 283 1272–1273. 10.1126/science.283.5406.1272 10084931

[B60] Petit O. (2010). “Social competition and conflict resolution,” in *Encyclopedia of Behavioral Neuroscience*, eds Koob G. F. Le Moal M. Thompson R. F. (Amsterdam: Elsevier), 276–281. 10.1016/B978-0-08-045396-5.00114-7

[B61] Pottinger T. (2008). The stress response in fish-mechanisms, effects and measurement. *Fish Welfare* 32–48. 10.1002/9780470697610

[B62] R Core Team. (2024). *R: a Language and Environment for Statistical Computing.* Vienna: R Foundation for Statistical Computing.

[B63] Rambo C. Mocelin R. Marcon M. Villanova D. Koakoski G. Abreu M. et al.. (2017). Gender differences in aggression and cortisol levels in zebrafish subjected to unpredictable chronic stress. *Physiol. Behav.* 171 50–54. 10.1016/j.physbeh.2016.12.032 28039073

[B64] Reddon A. Ruberto T. Reader S. (2021). Submission signals in animal groups. *Behaviour* 159 1–20. 10.1163/1568539X-bja10125

[B65] Reddon A. R. Dey C. J. Balshine S. (2019). Submissive behaviour is mediated by sex, social status, relative body size and shelter availability in a social fish. *Anim. Behav.* 155, 131–139. 10.1016/j.anbehav.2019.06.026

[B66] Ruberto T. Talbot J. Reddon A. (2020). Head up displays are a submission signal in the group-living daffodil cichlid. *Behav. Process.* 181:104271. 10.1016/j.beproc.2020.104271 33053419

[B67] Satoh S. Ota K. Awata S. Kohda M. (2019). Dynamics of sibling aggression of a cichlid fish in Lake Tanganyika. *Hydrobiologia* 832 201–213. 10.1007/s10750-018-3768-8

[B68] Scaia M. Cavallino L. Pandolfi M. (2020). Social control of spermatogenesis and steroidogenesis in cichlid fish: A comparative approach. *Reproduction* 159 R31–R43. 10.1530/REP-18-0650 31426026

[B69] Scaia M. Morandini L. Noguera C. Ramallo M. Somoza G. Pandolfi M. (2018). Fighting cichlids: Dynamic of intrasexual aggression in dyadic agonistic encounters. *Behav. Process.* 147 61–69. 10.1016/j.beproc.2017.12.015 29273550

[B70] Scaia M. Trudeau V. Somoza G. Pandolfi M. (2023). Fighting cichlids: An integrated multimodal analysis to understand female and male aggression in Cichlasoma dimerus. *Horm. Behav.* 148:105301. 10.1016/j.yhbeh.2022.105301 36623433

[B71] Sefc K. Brown A. Clotfelter. (2014). Carotenoid-based coloration in cichlid fishes. *Comp. Biochem. Physiol. A Mol. Integr. Physiol.* 173 42–51. 10.1016/j.cbpa.2014.03.006 24667558 PMC4003536

[B72] Soares M. Oliveira R. Ros A. Grutter A. Bshary R. (2011). Tactile stimulation lowers stress in fish. *Nat. Commun.* 2:534. 10.1038/ncomms1547 22086335

[B73] Sopinka N. Donaldson M. O’Connor C. Suski C. Cooke S. (2016). Stress indicators in fish. *Fish Physiol.* 35 405–462. 10.1016/B978-0-12-802728-8.00011-4

[B74] Taborsky M. (2016). “Cichlid fishes: a model for the integrative study of social behavior,” in *Cooperative Breeding in Vertebrates*, ed. Dickinson J. (Cambridge: Cambridge University Press), 272–293. 10.1017/CBO9781107338357

[B75] Teresa F. Gonçalves-de-Freitas E. (2011). Reproductive behavior and parental roles of the cichlid fish Laetacara araguaiae. *Neotrop. Ichthyol.* 9 355–362. 10.1590/S1679-62252011005000018

[B76] Tibbetts E. Pardo-Sanchez J. Weise C. (2022). The establishment and maintenance of dominance hierarchies. *Philos. Trans. R. Soc. B* 377:20200450. 10.1098/rstb.2020.0450 35000449 PMC8743888

[B77] Torrezani C. Pinho-Neto C. Miyai C. Sanches F. Barreto R. (2013). Structural enrichment reduces aggression in Tilapia rendalli. *Mar. Freshw. Behav. Physiol.* 46 183–190. 10.1080/10236244.2013.805053

[B78] Walker S. Trotter P. Swaney W. Marshall A. Mcglone F. (2017). C-tactile afferents: Cutaneous mediators of oxytocin release during affiliative tactile interactions? *Neuropeptides* 64 27–38. 10.1016/j.npep.2017.01.001 28162847

[B79] Wazlavek B. Figler M. (1989). Territorial prior residence, size asymmetry, and escalation of aggression in convict cichlids (*Cichlasoma nigrofasciatum Günther*). *Aggress. Behav.* 15 235–244. 10.1002/1098-2337(1989)15:3<235::AID-AB2480150305>3.0.CO;2-C

[B80] Wendelaar-Bonga S. (1997). The stress response in fish. *Physiol. Rev.* 77 591–625. 10.1152/physrev.1997.77.3.591 9234959

[B81] Wimberger P. (1992). Plasticity of fish body shape. The effects of diet, development, family and age in two species of Geophagus (Pisces: Cichlidae). *Biol. J. Linnean Soc.* 45 197–218. 10.1111/j.1095-8312.1992.tb00640.x

[B82] Wong M. Balshine S. (2010). The evolution of cooperative breeding in the African cichlid fish, Neolamprologus pulcher. *Biol. Rev.* 86 511–530. 10.1111/j.1469-185x.2010.00158.x 20849492

[B83] Young C. (2019). “Agonistic behavior,” in *Encyclopedia of Animal Cognition and Behavior*, eds Vonk J. Shackelford T. (Cham: Springer). 10.1007/978-3-319-47829-6_320-1

