# Supplementary material for: Body tactile stimulation intensifies appeasement behavior in a cichlid fish
Source: Front Behav Neurosci. 2026 Aug 20;20:1821679. doi: 10.3389/fnbeh.2026.1821679 (PMC13538113; doi:10.3389/fnbeh.2026.1821679)
Supplement: Supplementary file 1 [file Table_1.docx]

**Table S1.** Focal sampling with time instantaneous recording to quantify the submissive behaviour. The behaviour of the intruder (focal) fish during the fight was instantaneously recorded every 15 seconds for 30 minutes (120 intervals). A '1' was marked at the sample point in which the behaviour was observed, and a '0' was marked if it did not occur. The relative frequency was then taken as a score. The higher the score, the higher the behavioural intensity. Furthermore, we used an accumulated score by summing up the total of occurrences every 5 minutes and dividing it by 20, which is the number of intervals within 5 minutes. Herein, we show replicate 1 from the treatment without tactile stimulation as an example.

|  | **Minute 1** | | | | **Minute 2** | | | | **Minute 3** | | | | **Minute 4** | | | | **Minute 5** | | | |
| --- | --- | --- | --- | --- | --- | --- | --- | --- | --- | --- | --- | --- | --- | --- | --- | --- | --- | --- | --- | --- |
| **Behavioural unit** | **15s** | **30s** | **45s** | **60s** | **15s** | **30s** | **45s** | **60s** | **15s** | **30s** | **45s** | **60s** | **15s** | **30s** | **45s** | **60s** | **15s** | **30s** | **45s** | **60s** |
| Lateral orientation | 0 | 0 | 0 | 0 | 0 | 0 | 0 | 0 | 0 | 0 | 0 | 0 | 0 | 0 | 0 | 0 | 0 | 0 | 0 | 0 |
| Near Wall | 1 | 1 | 1 | 1 | 0 | 0 | 0 | 0 | 0 | 0 | 0 | 0 | 0 | 0 | 0 | 0 | 0 | 0 | 0 | 0 |
| Wall facing | 0 | 0 | 0 | 0 | 0 | 0 | 0 | 0 | 1 | 0 | 0 | 0 | 1 | 1 | 0 | 0 | 1 | 1 | 1 | 0 |
| Vertical orientation | 0 | 0 | 0 | 0 | 0 | 0 | 0 | 0 | 0 | 0 | 0 | 0 | 0 | 0 | 0 | 0 | 0 | 0 | 0 | 0 |
| Vertical orbital bar darkening | 1 | 1 | 1 | 1 | 1 | 0 | 0 | 0 | 0 | 0 | 0 | 0 | 0 | 0 | 0 | 0 | 0 | 0 | 0 | 0 |
| Midlateral spot darkening | 1 | 1 | 1 | 1 | 1 | 1 | 0 | 0 | 1 | 1 | 0 | 1 | 1 | 1 | 0 | 1 | 1 | 1 | 1 | 1 |
| Vertical body bars darkening | 1 | 1 | 1 | 0 | 0 | 0 | 0 | 0 | 0 | 0 | 0 | 0 | 0 | 0 | 0 | 0 | 0 | 0 | 0 | 0 |
| Tolerance | 0 | 0 | 0 | 0 | 0 | 0 | 0 | 0 | 0 | 0 | 0 | 0 | 0 | 0 | 0 | 0 | 0 | 0 | 0 | 0 |
| Fleeing | 0 | 0 | 0 | 0 | 0 | 0 | 0 | 0 | 0 | 0 | 0 | 0 | 0 | 0 | 0 | 0 | 0 | 0 | 0 | 1 |
|  | **Minute 6** | | | | **Minute 7** | | | | **Minute 8** | | | | **Minute 9** | | | | **Minute 10** | | | |
| **Behavioural unit** | **15s** | **30s** | **45s** | **60s** | **15s** | **30s** | **45s** | **60s** | **15s** | **30s** | **45s** | **60s** | **15s** | **30s** | **45s** | **60s** | **15s** | **30s** | **45s** | **60s** |
| Lateral orientation | 0 | 0 | 0 | 0 | 0 | 0 | 0 | 0 | 0 | 0 | 0 | 0 | 0 | 0 | 0 | 0 | 0 | 0 | 0 | 0 |
| Near Wall | 0 | 0 | 0 | 0 | 0 | 0 | 0 | 0 | 0 | 0 | 0 | 0 | 0 | 0 | 0 | 0 | 0 | 0 | 0 | 0 |
| Wall facing | 0 | 0 | 0 | 0 | 0 | 0 | 0 | 0 | 0 | 0 | 0 | 1 | 0 | 0 | 0 | 0 | 0 | 0 | 0 | 0 |
| Vertical orientation | 0 | 0 | 0 | 0 | 0 | 0 | 0 | 0 | 0 | 0 | 0 | 0 | 0 | 0 | 0 | 0 | 0 | 0 | 0 | 0 |
| Vertical orbital bar darkening | 0 | 0 | 0 | 0 | 0 | 0 | 0 | 0 | 1 | 0 | 0 | 0 | 0 | 0 | 0 | 0 | 0 | 0 | 0 | 0 |
| Midlateral spot darkening | 1 | 1 | 1 | 1 | 1 | 1 | 1 | 1 | 1 | 1 | 0 | 0 | 0 | 0 | 0 | 0 | 0 | 0 | 0 | 0 |
| Vertical body bars darkening | 0 | 0 | 0 | 0 | 0 | 0 | 0 | 0 | 0 | 0 | 0 | 0 | 0 | 0 | 0 | 0 | 0 | 0 | 0 | 0 |
| Tolerance | 0 | 0 | 0 | 0 | 0 | 0 | 0 | 0 | 0 | 0 | 0 | 0 | 0 | 0 | 0 | 0 | 0 | 0 | 0 | 0 |
| Fleeing | 1 | 1 | 0 | 0 | 1 | 1 | 0 | 0 | 0 | 0 | 0 | 0 | 1 | 0 | 0 | 0 | 0 | 0 | 0 | 0 |
|  | **Minute 11** | | | | **Minute 12** | | | | **Minute 13** | | | | **Minute 14** | | | | **Minute 15** | | | |
| **Behavioural unit** | **15s** | **30s** | **45s** | **60s** | **15s** | **30s** | **45s** | **60s** | **15s** | **30s** | **45s** | **60s** | **15s** | **30s** | **45s** | **60s** | **15s** | **30s** | **45s** | **60s** |
| Lateral orientation | 0 | 0 | 0 | 0 | 0 | 0 | 0 | 0 | 0 | 0 | 0 | 0 | 0 | 0 | 1 | 0 | 0 | 0 | 0 | 0 |
| Near Wall | 0 | 0 | 0 | 0 | 0 | 0 | 0 | 0 | 0 | 0 | 0 | 0 | 0 | 0 | 0 | 0 | 0 | 0 | 0 | 0 |
| Wall facing | 0 | 0 | 0 | 0 | 0 | 0 | 0 | 0 | 0 | 0 | 0 | 0 | 0 | 0 | 0 | 0 | 0 | 1 | 0 | 1 |
| Vertical orientation | 0 | 0 | 0 | 0 | 0 | 0 | 0 | 0 | 0 | 0 | 0 | 0 | 0 | 0 | 0 | 0 | 1 | 0 | 0 | 0 |
| Vertical orbital bar darkening | 0 | 0 | 0 | 0 | 0 | 0 | 0 | 0 | 0 | 0 | 0 | 0 | 0 | 1 | 1 | 0 | 0 | 0 | 0 | 0 |
| Midlateral spot darkening | 0 | 0 | 0 | 0 | 0 | 0 | 0 | 0 | 0 | 0 | 0 | 0 | 0 | 1 | 1 | 0 | 0 | 1 | 1 | 1 |
| Vertical body bars darkening | 0 | 0 | 0 | 0 | 0 | 0 | 0 | 0 | 0 | 0 | 0 | 0 | 0 | 1 | 1 | 0 | 0 | 0 | 1 | 1 |
| Tolerance | 0 | 0 | 0 | 0 | 0 | 0 | 0 | 0 | 0 | 0 | 0 | 0 | 0 | 0 | 0 | 0 | 0 | 0 | 0 | 0 |
| Fleeing | 0 | 0 | 0 | 0 | 0 | 0 | 0 | 0 | 0 | 0 | 0 | 0 | 0 | 0 | 0 | 0 | 0 | 0 | 0 | 0 |
|  | **Minute 16** | | | | **Minute 17** | | | | **Minute 18** | | | | **Minute 19** | | | | **Minute 20** | | | |
| **Behavioural unit** | **15s** | **30s** | **45s** | **15s** | **30s** | **45s** | **60s** | **15s** | **30s** | **45s** | **60s** | **60s** | **15s** | **30s** | **45s** | **60s** | **15s** | **30s** | **45s** | **60s** |
| Lateral orientation | 0 | 0 | 0 | 0 | 0 | 0 | 0 | 0 | 0 | 0 | 0 | 0 | 0 | 0 | 0 | 0 | 0 | 0 | 0 | 0 |
| Near Wall | 0 | 0 | 0 | 0 | 0 | 0 | 0 | 0 | 0 | 0 | 0 | 0 | 0 | 0 | 0 | 0 | 0 | 0 | 0 | 0 |
| Wall facing | 1 | 0 | 1 | 0 | 0 | 0 | 0 | 0 | 0 | 1 | 0 | 0 | 0 | 0 | 0 | 0 | 0 | 0 | 0 | 0 |
| Vertical orientation | 0 | 0 | 0 | 0 | 0 | 0 | 0 | 0 | 0 | 0 | 0 | 0 | 0 | 0 | 0 | 0 | 0 | 0 | 0 | 0 |
| Vertical orbital bar darkening | 0 | 0 | 0 | 0 | 0 | 0 | 0 | 0 | 0 | 0 | 0 | 0 | 0 | 0 | 0 | 0 | 0 | 0 | 0 | 0 |
| Midlateral spot darkening | 1 | 1 | 1 | 1 | 1 | 1 | 1 | 1 | 1 | 1 | 1 | 1 | 1 | 1 | 1 | 1 | 1 | 1 | 1 | 1 |
| Vertical body bars darkening | 1 | 1 | 1 | 1 | 1 | 1 | 1 | 1 | 1 | 1 | 0 | 1 | 1 | 1 | 1 | 1 | 1 | 1 | 1 | 1 |
| Tolerance | 0 | 0 | 0 | 0 | 0 | 0 | 0 | 0 | 0 | 0 | 1 | 0 | 0 | 0 | 0 | 0 | 0 | 0 | 0 | 0 |
| Fleeing | 0 | 0 | 0 | 0 | 0 | 0 | 0 | 0 | 0 | 0 | 0 | 1 | 0 | 0 | 0 | 0 | 0 | 0 | 0 | 0 |
|  | **Minute 21** | | | | **Minute 22** | | | | **Minute 23** | | | | **Minute 24** | | | | **Minute 25** | | | |
| **Behavioural unit** | **15s** | **30s** | **45s** | **60s** | **15s** | **30s** | **45s** | **60s** | **15s** | **30s** | **45s** | **60s** | **15s** | **30s** | **45s** | **60s** | **15s** | **30s** | **45s** | **60s** |
| Lateral orientation | 0 | 0 | 0 | 0 | 0 | 0 | 0 | 0 | 0 | 0 | 0 | 0 | 1 | 0 | 1 | 0 | 1 | 1 | 0 | 0 |
| Near Wall | 0 | 0 | 0 | 0 | 0 | 0 | 0 | 0 | 0 | 0 | 0 | 0 | 1 | 1 | 1 | 1 | 1 | 1 | 1 | 0 |
| Wall facing | 0 | 0 | 0 | 0 | 0 | 0 | 0 | 1 | 0 | 0 | 0 | 0 | 0 | 0 | 0 | 0 | 0 | 0 | 0 | 0 |
| Vertical orientation | 0 | 0 | 0 | 0 | 0 | 0 | 0 | 0 | 0 | 0 | 1 | 1 | 0 | 0 | 0 | 0 | 0 | 0 | 0 | 0 |
| Vertical orbital bar darkening | 0 | 0 | 0 | 0 | 0 | 0 | 0 | 0 | 0 | 0 | 1 | 1 | 1 | 0 | 0 | 1 | 0 | 0 | 0 | 0 |
| Midlateral spot darkening | 1 | 1 | 1 | 1 | 1 | 1 | 1 | 1 | 1 | 1 | 1 | 1 | 0 | 0 | 0 | 1 | 0 | 0 | 0 | 0 |
| Vertical body bars darkening | 1 | 1 | 0 | 0 | 0 | 1 | 1 | 1 | 0 | 0 | 0 | 0 | 0 | 0 | 0 | 0 | 0 | 0 | 0 | 0 |
| Tolerance | 0 | 0 | 0 | 0 | 0 | 0 | 0 | 0 | 0 | 0 | 0 | 0 | 0 | 0 | 1 | 0 | 0 | 0 | 0 | 0 |
| Fleeing | 0 | 0 | 1 | 1 | 0 | 0 | 0 | 0 | 0 | 0 | 0 | 0 | 0 | 0 | 0 | 0 | 0 | 0 | 0 | 0 |
|  | **Minute 26** | | | | **Minute 27** | | | | **Minute 28** | | | | **Minute 29** | | | | **Minute 30** | | | |
| **Behavioural unit** | **15s** | **30s** | **45s** | **60s** | **15s** | **30s** | **45s** | **60s** | **15s** | **30s** | **45s** | **60s** | **15s** | **30s** | **45s** | **60s** | **15s** | **30s** | **45s** | **60s** |
| Lateral orientation | 0 | 0 | 0 | 0 | 1 | 1 | 1 | 1 | 1 | 1 | 1 | 1 | 1 | 1 | 1 | 1 | 1 | 1 | 1 | 1 |
| Near Wall | 0 | 0 | 0 | 0 | 0 | 0 | 0 | 0 | 0 | 0 | 0 | 0 | 0 | 0 | 0 | 0 | 0 | 0 | 0 | 0 |
| Wall facing | 0 | 0 | 0 | 0 | 0 | 0 | 0 | 0 | 0 | 0 | 0 | 0 | 0 | 0 | 0 | 0 | 0 | 0 | 0 | 0 |
| Vertical orientation | 0 | 0 | 0 | 0 | 0 | 0 | 0 | 0 | 0 | 0 | 0 | 0 | 0 | 0 | 0 | 0 | 0 | 0 | 0 | 0 |
| Vertical orbital bar darkening | 0 | 0 | 0 | 0 | 0 | 0 | 0 | 0 | 0 | 0 | 0 | 0 | 0 | 0 | 0 | 0 | 0 | 0 | 0 | 0 |
| Midlateral spot darkening | 1 | 1 | 0 | 0 | 1 | 1 | 1 | 0 | 0 | 0 | 0 | 0 | 0 | 0 | 0 | 0 | 0 | 0 | 0 | 0 |
| Vertical body bars darkening | 0 | 0 | 0 | 0 | 0 | 0 | 0 | 0 | 0 | 0 | 0 | 0 | 0 | 0 | 0 | 0 | 0 | 0 | 0 | 0 |
| Tolerance | 0 | 0 | 0 | 0 | 0 | 0 | 0 | 0 | 0 | 0 | 0 | 0 | 0 | 0 | 0 | 0 | 0 | 0 | 0 | 0 |
| Fleeing | 0 | 0 | 0 | 0 | 0 | 0 | 0 | 0 | 0 | 0 | 0 | 0 | 0 | 0 | 0 | 0 | 0 | 0 | 0 | 0 |
|  | **Scores** | | | | | | | | | | | | | | | | | | | |
| **Behavioural unit** | **Score** | | | | | | | | **Time interval** | | | | **Accumulated score** | | | | | | | |
| Lateral orientation | 0.18 | | | | | | | | 00 - 05 min | | | | 1.75 | | | | | | | |
| Near Wall | 0.09 | | | | | | | | 06 - 10 min | | | | 0.85 | | | | | | | |
| Wall facing | 0.11 | | | | | | | | 11 - 15 min | | | | 0.85 | | | | | | | |
| Vertical orientation | 0.03 | | | | | | | | 16 - 20 min | | | | 2.10 | | | | | | | |
| Vertical orbital bar darkening | 0.10 | | | | | | | | 21 - 25 min | | | | 1.95 | | | | | | | |
| Midlateral spot darkening | 0.58 | | | | | | | | 26 - 30 min | | | | 1.10 | | | | | | | |
| Vertical body bars darkening | 0.25 | | | | | | | |  | | | |  | | | | | | | |
| Tolerance | 0.02 | | | | | | | |  | | | |  | | | | | | | |
| Fleeing | 0.08 | | | | | | | |  | | | |  | | | | | | | |
